# Recommendations for the Treatment of Invasive Fungal Infections in Hematological Malignancies: A Critical Review of Evidence and Turkish Expert Opinion (TEO-1)

**DOI:** 10.4274/tjh.2014.0103

**Published:** 2014-06-10

**Authors:** Hamdi Akan, Şeniz Öngören Aydın, Neşe Saltoğlu, Atahan Çağatay, Halis Akalın, Mutlu Arat, Rıdvan Ali, Sevgi Kalayoğlu-Beşışık, A. Muzaffer Demir

**Affiliations:** 1 Ankara University Faculty of Medicine Cebeci Campus, Department of Hematology, Ankara, Turkey; 2 İstanbul University Cerrahpaşa Faculty of Medicine, Department of Hematology, İstanbul, Turkey; 3 Istanbul University Cerrahpasa Faculty of Medicine, Department of Infectious Diseases, İstanbul, Turkey; 4 İstanbul University Istanbul Faculty of Medicine, Department of Infectious Diseases and Clinical Microbiology, İstanbul, Turkey; 5 Uludağ University Faculty of Medicine, Department of Infectious Diseases and Clinical Microbiology, Bursa, Turkey; 6 Florence Nightingale Hospital, Hematology Unit, İstanbul, Turkey; 7 Uludağ University Faculty of Medicine, Department of Hematology, Bursa, Turkey; 8 Trakya University Faculty of Medicine, Department of Hematology, Edirne, Turkey

**Keywords:** invasive fungal infection, Antifungal treatment, Evidence, Hematological malignancies

## Abstract

The introduction of novel antifungal agents for the treatment of invasive fungal disease in hematological malignancies and also changing treatment strategies have had a great impact in managing affected patients. The medical literature includes some important clinical studies that are being used as evidence for guidelines. The problem with these studies and the guidelines is that they are not very easy to interpret, they include controversial issues, and they are not easy to apply to every patient or country. This paper was designed to critically show the main problems associated with these approaches and provide important information that will help Turkish doctors to adopt them in daily clinical practice.

## Abstract (tr)

Hematolojik malignitelerde yeni antifungal ajanların invazif fungal infeksiyonların tedavisine sunulması ve tedavi stratejilerindeki değişmeler, bu hastaların tedavisini önemli şekilde etkilemiştir. Tıp literatüründe halen kılavuzlara kaynak olan bazı önemli klinik çalışmalar vardır. Ancak gerek bu çalışmalar gerekse kılavuzların en önemli sorunları günlük tıp pratiğine uyarlamadaki güçlükler, tartışmalı konular içermeleri ve her hasta ya da ülkeye uyarlama güçlüğü olmasıdır. Bu yazıda bu yaklaşımlarla ilgili önemli sorunlar eleştirel bir bakış açısı ile gözden geçirilmiş ve Türkiyede doktorların günlük tıp pratiklerine uyarlamaları konusunda bilgiler verilmiştir.

## INTRODUCTION

Invasive fungal infections (IFIs) have emerged as a serious problem affecting morbidity and mortality in the management of patients with hematological malignancies. The continuous introduction of novel approaches and medications in this area influences the clinical behavior of physicians. In order to arrive at a consensus decision, various guidelines have been published and updated. These guidelines make recommendations based on evidence from certain important published articles. However, discordance between clinical research and medical practice comes into question and the implementation of these guidelines or the adaptation of them according to the local regulations of different countries may be troublesome. From this point of view, a critical review of evidence was undertaken by the experts on IFI management in Turkey. We performed a critical appraisal of some studies that have been unconditionally accepted, the results of which have been implemented until today. This paper includes appraisals of 7 key articles on IFI. These articles have been divided into 2 groups according to their subjects. The first group, i.e. the first 3 studies, is generally called the “Walsh studies” [1,2,3] and the efficacies of various antifungal agents used in empirical treatment are compared in these studies. As a standard approach, empirical antifungal treatment is given for the early treatment of fungal infections in neutropenic patients with persistent fever without clinical findings. Although the main aim in these studies is to determine the differences in efficacy of antifungal agents used in empirical treatment, the differences in side effects come to the forefront. The 4 studies in the second group are strategy studies [4,5,6,7]. The use of “prolonged fever/febrile neutropenia” as the sole evidence for fungal infection in empirical treatment has begun to be questioned and the requirement of using diagnostic tools such as high-resolution computed tomography (HRCT) and galactomannan (GM) revealed the probability of the substitution of the empirical approach with new strategies like preemptive treatment or diagnostic-driven treatment. These 4 studies investigate and compare the benefits of empirical versus preemptive approaches as early treatment strategies for IFIs. Outlines of the clinical trials included in this review are found in Table 1.

**Efficacy Studies**

In the 3 studies in the first group, Walsh and his colleagues used treatment success as the primary endpoint [1,2,3]. They defined a composite score involving 5 criteria: 7-day survival after the initiation of the studied drug, resolution of fever during the neutropenic period, successful treatment of baseline fungal infection, absence of breakthrough fungal infections while receiving the studied drug or within 7 days after treatment, and no early withdrawal of the studied drug due to toxicity or lack of efficacy.

First Study: “Liposomal amphotericin B for empirical therapy in patients with persistent fever and neutropenia” by Walsh et al. [1] 

Author: Şeniz Öngören Aydın, Associate Professor, MD 

Previously, 2 randomized placebo-controlled studies on empirical antifungal treatment showed that treatment with conventional amphotericin B deoxycholate decreased the frequency of proven IFIs [8,9]. The newly developed lipid formulations of amphotericin B led to a decrease in dose-limiting nephrotoxicity and infusion-related acute toxic effects. 

Both preclinical and open-label phase 1 and 2 studies demonstrated that liposomal amphotericin B (LAmB) is more efficient and less nephrotoxic than conventional amphotericin B in the treatment of invasive fungal infections (disseminated candidiasis and invasive pulmonary aspergillosis) [10,11,12,13,14,15].

**Critical Review of the Study**

This study comparing empirical LAmB with conventional amphotericin B in neutropenic patients with persistent fever is one of the studies that is cited the most among the papers on antifungal treatment in febrile neutropenic patients. The use of masking is the most important strength of this study.

There were 4 important studies that compared LAmB with conventional amphotericin B in the empirical treatment of febrile neutropenic patients [1,16,17,18]. The results of these studies are similar to the results obtained in the Walsh study. 

The most important contribution of this study in the follow-up and evaluation of febrile neutropenic patients is the defining of the composite score for determining the success of antifungal treatment used. Fever can occur for many reasons other than infection, and treatment success was not based on the sole use of resolution of fever in this study, an aspect of critical importance also acknowledged by the guidelines.

Regarding statistical significance, the sample size calculated before the study was attained in both groups. However, inclusion and analysis of pediatric and adult patients together (age range: 2-80 years) constitutes one of the weak points of the study. 

Regarding risk categories, the groups were quite heterogeneous, neutropenia duration was not taken into consideration, patients with allogeneic stem cell transplantation (alloSCT) and autologous stem cell transplantation (ASCT) were classified in the same risk group, and, although there were patients who underwent alloSCT, the duration of neutropenia being short for the development of deep fungal infections was intriguing. Additionally, although the patients who underwent stem cell transplantation were considered as high-risk patients, the total number of patients in the high-risk group was low (i.e. inconsistency in the number of patients with high risk). 

Although it was mentioned that the groups were comparable regarding antibacterial, antifungal, and antiviral treatment at baseline, the onset of antifungal treatment and time of antibiotic modification/addition (glycopeptides) was not mentioned. 

Additionally, there is no information on the outcome of patients with candidemia, who were of equal number in both groups at the beginning of the study. 

Although aggressive interventions (blood culture, bronchoalveolar lavage, percutaneous needle aspiration, and biopsy) were carried out to identify the etiology of fungal infections, most patients were diagnosed to have suspected fungal pneumonia, as in daily practice. The frequency of infusion-related toxicities was lower in patients using LAmB; data showing the results of >7000 infusions support the statistical power of the study.

In 2002, Cagnoni reanalyzed the 103 patients who underwent alloSCT who were included in the study of Walsh [19]. Nephrotoxicity (p<0.001), breakthrough infections (p<0.05), and dose reduction requirements (p<0.001) were lower in the LAmB group. From the pharmacoeconomic point of view, although the drug was cheap, the prolonged hospital stays, dialysis requirements, and other supportive treatments of patients with nephrotoxicity led to a significant increase in cost in the conventional amphotericin B group (p<0.001).

Second Study: “Voriconazole compared with liposomal amphotericin B for empirical antifungal therapy in patients with neutropenia and persistent fever” by Walsh et al. [2] 

Author: Neşe Saltoğlu, Professor, MD 

This prospective, randomized, international multicenter, open-label study was based on the hypothesis that voriconazole is as efficacious and safe as LAmB in empirical antifungal treatment. 

The US Food and Drug Administration (FDA) reviewed the study and some issues were pointed out. Although the authors stated that they would present the overall stratified rate of response in terms of a 5-part composite end score, they presented an unstratified analysis in their report. As the patients were stratified according to the degree of risk of fungal infection and antifungal prophylaxis at randomization, stratified analysis rather than composite analysis would have been better in analyzing the data. The study did not meet the predefined primary endpoint. Additionally, multiple statistical comparisons were undertaken that might have increased the risk of false positive results. Therefore, the FDA recommended setting the statistical 

significance level at p<0.001 rather than at p<0.05 [20].

Marr wrote a letter to the editor in response and indicated that the inclusion of patients with renal insufficiency, but not those with hepatic failure, might have led to negative results regarding LAmB. Additionally, it was stated that the use of a composite endpoint in the evaluation was not suitable and analysis of each component individually would have been better [21]. Additionally, Petrikkos and Skiada suggested that the use of a composite endpoint score might have masked the individual controversial results related to safety and efficacy [22].

**Critical Review of the Study**

Regarding the 5-part composite score, except for voriconazole providing more beneficial results regarding the development of breakthrough infections, the effects of voriconazole were similar to or lower than those of LAmB. Additionally, there was no important difference between the 2 groups regarding the rate of breakthrough infections (98% versus 95%). As the data were analyzed by composite analysis, it is not possible to interpret the results clearly. 

Both drugs had similar side effects; however, voriconazole was associated with a lower rate of infusion-related toxicity and nephrotoxicity. 

In conclusion, the interpretation of study results according to risk groups defined at the beginning of the study would have allowed a better evaluation in this randomized controlled study comparing the effects of voriconazole with LAmB. Although breakthrough infections were observed at a lower rate in patients using voriconazole, the design of the analysis led to controversial results. 

Third Study: “Caspofungin versus liposomal amphotericin B for empirical antifungal therapy in patients with persistent fever and neutropenia” by Walsh et al. [3]

Author: Atahan Çağatay, Professor, MD 

Side effects, unpredictable pharmacokinetics, and limited activity may become important clinical problems while using amphotericin B and triazoles. Therefore, the efficacy of a new class of antifungal agents in empirical treatment, echinocandins, was evaluated. Caspofungin, an echinocandin, is known to be effective against Candida and Aspergillus species and has been approved to be used for esophageal candidiasis, candidemia, and other Candida infections and in patients intolerant to other antifungal treatments or in treatment for refractory Aspergillus infections. Therefore, the efficacy and safety of LAmB and caspofungin in the empirical treatment of patients with neutropenia and persistent fever was compared in this study. 

**Critical Review of the Study**

The most striking result obtained in this study was the significant rate of unexpected findings in patients with baseline Aspergillus spp. infection. The response rate of 8.3% reported in this study is the lowest response rate recorded for LAmB in Aspergillus infections. This situation raises the question of whether there was a problem in the distribution of patient groups. Again, the rate of resolution of baseline fungal infection was found to be lower in the LAmB group (25.9%), while the corresponding rate was 66.7% when comparing LAmB with voriconazole and 81.8% when comparing conventional amphotericin B with LAmB.

Although it was a ‘noninferiority’ study, the general survival rate of patients with baseline fungal infection (mostly invasive aspergillosis) was lower and the results were worse among patients treated with LAmB. The starting dose of LAmB was 3 mg/kg in the study; in the case of no clinical response, the treatment dose was increased to 5 mg/kg/day after 5 days. In clinical practice, LAmB can be used at 5 mg/kg/day in the treatment of invasive aspergillosis. In this study, it was stated that a suboptimal preloading dose (3 mg/kg instead of 5 mg/kg) might have led to efficacy problems in patients with baseline invasive aspergillosis. Therefore, it was thought that estimation of the general survival rate with Kaplan–Meier analysis, after excluding patients with baseline fungal infections, would give clearer results. The importance of initiating early antifungal treatment was stressed elsewhere related to this study [23]. The predominance of fungal pathogens with natural resistance is intriguing [24,25,26,27,28].

**B. Strategy Studies**

Fourth Study: “Galactomannan and computed tomography-based preemptive antifungal therapy in neutropenic patients at high risk for invasive fungal infection: a prospective feasibility study” by Maertens et al. [4]

Author: Halis Akalın, Professor, MD 

Initiation of empirical antifungal treatment in neutropenic patients with fever persisting for 5-7 days and IFI risk despite wide-spectrum antibiotic treatment has been part of standard care. However, the fact that fever is unrelated to fungal infection in the majority of these patients has started debates about the use of unnecessary antifungal treatment in some of these patients. On the other hand, new methods that can be used in IFI diagnosis, such as GM, β-D-glucan, and HRCT, have been introduced. 

This study performed by Maertens et al. evaluated the applicability of a preemptive approach based on the assessment of serum GM and computed chest tomography in high-risk neutropenic patients, the change in the number of patients receiving antifungal treatment, and the advantages and disadvantages of this approach to empirical antifungal treatment regarding prognosis.

A letter to the editor by de Pauw was published in Clinical Infectious Diseases [29]. As the value of the conventional empirical strategy against Aspergillus spp. is controversial and may lead to everlasting debates on the antifungal agent that should be chosen, de Pauw found it inconvenient to perform randomized comparative studies on this topic. He stated that a well-defined and autopsy-controlled study would be more beneficial in answering many questions regarding the reliability of diagnostic tests and whether it is possible to use a preemptive approach in nonneutropenic patients. He emphasized that starting antifungal treatment in neutropenic patients who show no response to antibiotic treatment is no longer valid, and there are various risk factors besides neutropenia. 

He also highlighted that the success of preemptive strategies is associated with meticulously close follow-up of the patient as well as the use of timely and repeatable reliable diagnostic tests. He stated that preemptive treatment can alter antifungal treatment approach in immunosuppressed patients. 

**Critical Review of the Study**

This study opened a door to a new approach that could reduce the use of unnecessary antifungal treatment in patients with febrile neutropenia. With this study, fever persisting despite wide-spectrum antibiotic treatment being the sole reason for antifungal treatment is questioned in real terms. Beyond a doubt, novel diagnostic tests and advances in radiology have had important roles in reaching this point.

This study, from another point of view, showed that earlier diagnosis of patients with invasive pulmonary aspergillosis without fever or fever with other causes is possible by using GM.

Although the study not being a randomized comparative trial, having a small sample size, and lacking an assessment of GM in bronchoalveolar lavage can be considered as weak points, this study is a key publication to generate further hypotheses.

Fifth Study: “Empirical versus preemptive antifungal therapy for high-risk, febrile, neutropenic patients: a randomized, controlled trial” by Cordonnier et al. [5]

Author: Mutlu Arat, Professor, MD

In this study, the researchers compared the empirical and preemptive strategies in the treatment of probable IFI cases in febrile neutropenia patients with persistent fever. 

de Pauw and Donnely wrote a detailed editorial about this study [30]. Although they criticized the philosophy of targeting overall survival and other factors, they shared the opinion that this study clearly underlined the importance of using the right treatment at the correct time. In particular, they cited studies that used GM antigenemia and thoracic computerized tomography more widely and more efficiently, and they stated that lack of autopsy findings is a limitation of the study. Although the study made an overwhelming impression in the infection world, it was destined to fall 2-3 years behind regarding its timing. 

In a letter to the editor written by Marr et al., antifungal prophylaxis not being standard and, particularly, the development of IFI in 5 patients who did not receive prophylaxis in the induction group were stated to support azole prophylaxis [31]. They underlined that the clinical indicators in the preemptive treatment group were not early but rather late findings, and this was stated as the reason for observing more frequent IFIs in this group. They highlighted that sensitive tests are required for prediction, and that the currently used tests reveal existence rather than nonexistence more precisely; they also put forward the idea of de-escalation treatment. Another critique came from Stefani et al. [32]. They criticized the inclusion of patients with ASCT and lymphoma in the study group and they stated that they were stunned by the exclusion of alloSCT cases. While prophylaxis weakness was verbalized, the requirement of closer microbiological follow-up was stressed, and the inclusion of a patient in shock was found to be questionable from an ethical point of view. The safety of the environment was not reported. 

**Critical Review of the Study**

The study was criticized both by the authors of the study and by authors writing letters to the editor. Considering survival as the aim of the study was deemed challenging and the nonhomogeneity of the comparison groups was criticized. Inclusion of ASCT and lymphoma cases with rare IFI frequency and exclusion of alloSCT cases with a high probability of IFI was intriguing. Toxicity, iatrogenic immunosuppression, and the probability of graft-versus-host disease in alloSCT cases might have intimidated the authors. Another common subject of criticism is the standardization of prophylaxis and close microbiological follow-up policy. Despite all these points, except for induction and probable alloSCT cases, the preemptive approach could be a more rational and cost-effective treatment with close follow-up, along with the evolving serological and imaging techniques

The point that I consider crucial in all febrile neutropenia studies is putting emphasis on the duration of neutropenia while failing to emphasize the kinetics of neutropenia. For clinicians and transplant physicians, the most important factor in fever control is neutrophil recovery. However, in the presently discussed study, although deep neutropenia seems to suggest a high risk, there is a borderline risk group with absolute neutropenia with a median duration of 10-12 days in the consolidation and ASCT group, and an induction group with absolute neutropenia with a median duration of 26 days. Considering the mean as 18 days and treating this patient population as a homogeneous group and classifying them into the same risk group is not a rational approach. 

In both ablative and nonablative transplantation, the course of transplant and survival is poor in febrile neutropenic attacks associated with the development of pneumonia and IFI in the first week. Apart from the depth of neutropenia at the time of febrile neutropenia, a second definition of “neutropenia duration after fever” should also be established. The following 3 intervals should be defined clearly and shared in the statistics of further studies: the time between the development of fever to the time to neutrophil recovery to >0.5x10^9^/L, the time from the onset of antifungal treatment to the recovery of neutrophils, and the time between resolution of fever and neutrophil recovery (this datum may be categorically “positive or negative”). In this regard, data on febrile neutropenia can be interpreted more clearly, and not only the depth and duration of neutropenia but also the impact of febrile neutropenia in the kinetics of neutrophil recovery will be exactly underlined. My belief is that, according to the timing of febrile neutropenia attack within the intervals mentioned above, the empirical or the preemptive approach can be used, especially for patients receiving induction chemotherapy and alloSCT. However, as kinetic data are not available in detail, it is not possible to deduce the implication of this for our current treatment schemes. 

Sixth Study: “Clinically driven diagnostic antifungal approach in neutropenic patients: a prospective feasibility study” by Girmenia et al. [6]

Author: Rıdvan Ali, Professor, MD 

Besides being an expensive method, serum GM screening (as a preemptive strategy) in neutropenic patients is time-consuming and not easy to interpret. This study was performed to determine the feasibility of a clinically driven diagnostic strategy with late GM screening. 

**Critical Review of the Study**

This study is valuable in that the same diagnostic criteria and tools were used in all study centers. Due to the difficulties of using GM in diagnosis, a first evaluation was made without using GM and HRCT; in comparison, clinically driven GM and HRCT use was found to be more efficient. Controversial results have been obtained in studies that compared the empirical approach with the diagnostic-driven approach, and this study contributed to the literature in favor of the diagnostic-driven approach. However, although the empirical approach was efficient in high-risk patients, and especially in the study of Cordonnier et al. [5], no such difference was noted in this study. The lack of autopsy-proven fungal infections is a limitation of the study; besides, a patient with mucormycosis died before treatment initiation by this approach. Nevertheless, this study suggests that the clinically driven diagnostic approach, supported primarily by radiological evaluation and subsequent GM results, can be efficient. 

Seventh Study: “The use and efficacy of empirical versus pre-emptive therapy in the management of fungal infections: the HEMA e-Chart Project” by Pagano et al. [7] 

Author: Sevgi Kalayoğlu-Beşışık, Professor, MD

This study was an observational study that included various acute or chronic hematological malignancies. They were managed by using a standard diagnostic approach in different centers. The efficacy of preemptive treatment versus empirical treatment was compared. The authors stated that although the empirical approach can lead to overtreatment, in specific patient groups (acute myeloid leukemia [AML] remission induction) it could decrease mortality.

**Critical Review of the Study**

This study included Hodgkin lymphoma, non-Hodgkin lymphoma, multiple myeloma, and myelodysplastic syndrome patients with different risk factors (severity and duration of mucositis, duration and severity of neutropenia, etc.). Efficacy of treatment (clinical course) was not reported clearly. 

In a study including patients who receive antifungal treatment, the choice of the antifungal agents that will be used in the empirical or preemptive treatment will depend on the type of antifungal prophylaxis. The death rate being high in the preemptive treatment group can be associated with the more common use of this method in probable or real IFI cases rather than delayed treatment [33,34].

**Experts’ Opinion Based on the Evidence**

In consideration of these studies together, the most important problem is the difficulty in comparing them because of different designs, methodologies, approaches, and patient selection criteria. As the Walsh studies included in this paper used the same methodology, they are more comparable; however, they were criticized because of methodological errors (selection of composite endpoints) and problems in patient inclusion criteria. Nevertheless, the results of these studies demonstrate that using LAmB and echinocandin (caspofungin) is a valid approach in empirical treatment. However, voriconazole was not approved for empirical use as it cannot reach the expected target. 

Although there was evidence supporting preemptive treatment in previous studies that evaluated treatment approaches, subsequent studies indicated that the empirical treatment approach still remains valid, especially in high-risk patients like those undergoing induction chemotherapy for AML. 

**Certain points stand out in the adaptation of these studies to Turkey:**

1. The efficacy data presented here are not in favor of conventional amphotericin B in empirical antifungal treatment. Additionally, a large randomized trial comparing voriconazole with conventional amphotericin B in invasive aspergillosis also showed that conventional amphotericin B is inferior in this setting [35]. Based on these studies, conventional amphotericin B is not recommended in the guidelines. Therefore, the scientific validity of the recommendations of the Healthcare Implementation Notification (Turkish: Sağlık Uygulama Tebliği) is questionable. In particular, patients at high risk for nephrotoxicity, such as those undergoing stem cell transplantation, should not use conventional amphotericin B.

2. In Turkey, drug choice in routine medical practice and drug licenses are aligned with the existing scientific data and it can be concluded that there is no major problem in this respect.

3. In empirical treatment, the use of LAmB is the valid approach, and caspofungin can be an alternative in first-line treatment.

4. Voriconazole is the drug of choice in suspected or documented Aspergillus infections; however, LAmB can also be used.

5. The preemptive treatment approach is more convenient in Turkey for several reasons, including the reduction of antifungal drug use, toxicity, and the costs of care. However, as timely and appropriate access to diagnostic tools is limited in most centers, the application of preemptive therapy is far from being as broad as that in the publications and radiological modalities generally come into prominence. In addition, the demonstration of increased mortality in certain risk groups with preemptive treatment raises some concerns about this approach. Therefore, unless new evidence emerges, initiation of treatment with the empirical approach, especially in patients in high-risk groups like those with acute leukemia induction, and modification of treatment according to the information obtained from diagnostic testing will be more reliable and valid for Turkey.

The review of these papers by Turkish experts showed us that, although there has been major progress in this area, the globalization and generalization of these approaches still need more study, and glocalization (globalization + localization) remains a valid approach in different countries.

## Figures and Tables

**Table 1:**
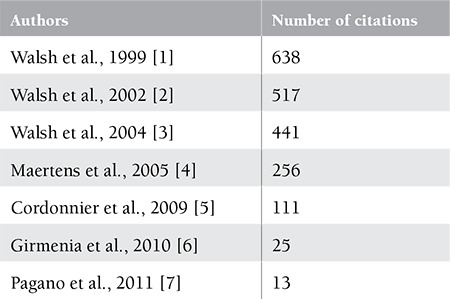
Number of citations of the articles(31 December 2013).

**Table 2:**
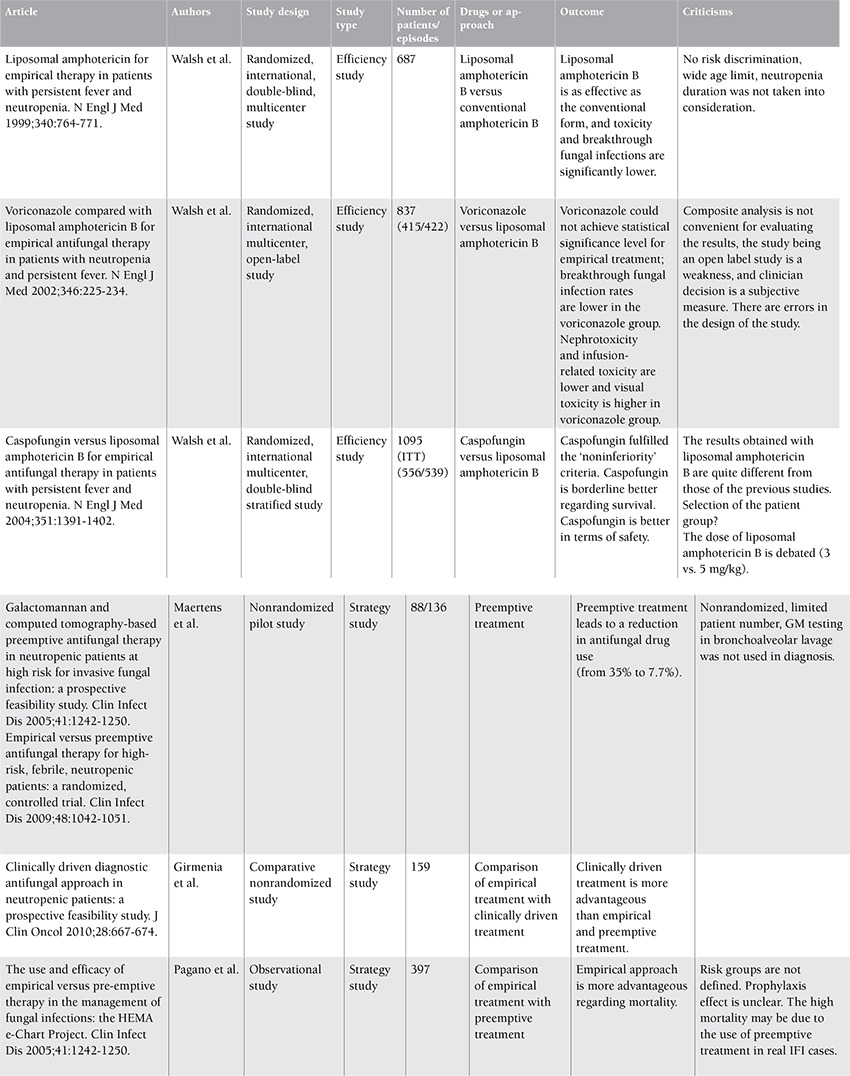
General characteristics of pivotal studies in the treatment of invasive fungal infections in hematological malignancies.
